# Morphology‐Dependent Sensitivity of Chitosan‐Based Sensing Materials for the Colorimetric Detection of β‐Glucuronidase From Pathogenic *E. coli*

**DOI:** 10.1002/mabi.70212

**Published:** 2026-07-08

**Authors:** Kawaljit Kaur, Kinyanjui Stephanie, Nthiga Esther, Douglas Onyancha, Holger Schönherr

**Affiliations:** ^1^ Physical Chemistry I Research Center of Micro‐ and Nanochemistry and (Bio) Technology Department of Chemistry and Biology School of Science and Technology University of Siegen Siegen Germany; ^2^ Department of Chemistry Dedan Kimathi University of Technology Nyeri Kenya

**Keywords:** bacteria detection, beads, chitosan, *E. coli*, film, scaffold, β‐Gus enzyme

## Abstract

To address the global threat of increasing antimicrobial resistance, the investigation and development of rapid identification and detection methods for bacteria and infection sensing approaches have become major areas of research. Expanding on earlier work on enzyme‐sensitive biopolymer‐based autonomous sensing materials, the impact of different morphologies and concomitantly surface‐to‐volume ratio on the sensitivity of the detection of the enzyme β‐glucuronidase (β‐Gus) from pathogenic and non‐pathogenic *E. coli* was unraveled. The enzymatic hydrolysis of microbeads and freeze‐dried scaffolds was compared to that of neat films equipped by post‐fabrication modification with 4‐methylumbelliferyl‐β‐D‐glucuronide hydrate (MUG) as a β‐Gus reporting functionality. Fluorescence spectroscopy and in vitro tests with planktonic MACH‐1 and NCTC strains revealed that the scaffolds outperformed the films with a close to ten‐fold increased sensitivity, while the beads showed an enhanced rate of only a factor of 2.4. For an observation time of 60 min, the scaffold, beads, and film exhibited a limit of detection (LOD) of 3.3, 13.4, and 17.5 nm, respectively. These results support the hypothesis that increased surface‐to‐volume ratios enhance the sensitivity, but also point to the role of surface porosity and accessibility of enzyme‐labile sites in swollen materials, which may be compromised due to the fabrication process.

## Graphical abstract

The impact of different morphologies and surface‐to‐volume ratio on the sensitivity of the detection of the enzyme β‐glucuronidase from *E. coli* using chitosan‐based enzyme‐sensitive autonomous sensing materials is unraveled. Using freeze‐drying, materials with a close to ten‐fold increased sensitivity are fabricated. The improved performance is attributed to an increased surface‐to‐volume ratio and enhanced porosity.

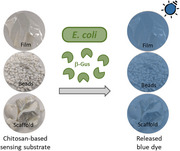

## 1 Introduction

The discovery of antibiotics in the last century resulted in a significant advancement in modern medicine [1]. It resulted in clearing most pathogenic bacteria, hence saving millions of lives and dramatically reducing mortality rates [2]. However, the continuous use and misuse of these antibiotics not only in medicine but also in agriculture has resulted in the development of AMR [3, 4]. AMR can be defined as the ability of microorganisms to tolerate and resist antimicrobial treatments, posing a major global health threat by making infections harder to treat. In 2021, 4.71 million deaths could be linked to bacterial AMR [5]. The World Health Organization (WHO) projects that this number may increase to 10 million annually by 2050 [6]. *Escherichia coli* (*E. coli*) remains among the top priority pathogens listed by the WHO [7]. This is because it has continued to jeopardize many of the advancements of contemporary medicine with consequences, such as longer hospitalization, significant constraints on healthcare costs, high morbidity and mortality rates, having a major impact, especially of susceptible communities, children, and even those with compromised immunity [8, 9, 10]. The detection of β‐Gus serves as a biomarker for bacterial presence, particularly *E. coli*.

The battle against AMR has been continuous, with the WHO directing investigations of new treatment methods against bacteria [11]. The need to not only find new antimicrobially active compounds but also to develop methods to slow down the build‐up and rate of resistance, or even stop the rapid development of antibiotic resistance by pathogenic microorganisms, is growing as AMR rises in many parts of the world. AMR mitigation requires early and accurate detection of bacterial infections. Early detection facilitates targeted antibiotic use, thereby reducing unnecessary broad‐spectrum antibiotic administration. Therefore, there is a need to design and develop simple, rapid, accurate, and selective detection methods to support efforts in combating AMR [12]. Conventional approaches that have been used for the detection of bacteria, such as those based on clinical signs, white blood cells count, tissue biopsies, wound swabs for culturing on agar plates, biological diagnostic methods such as next generation sequencing and PCR (polymer chain reactions) [13, 14, 15, 16] have proven to have some drawbacks such as, time consuming, labor intensive, need highly skilled personnel, require specialized equipment, have limited sensitivity and selectivity. Those that involve the disruption of the wound for a swab sample interfere with the healing and could result in further complications, such as necrosis [17]. Furthermore, contemporary methods that have proven reliable, rapid, selective and accurate rely according to the literature on the use of equipment, such as optical endomicroscopy, mass spectrometry and confocal laser scanning fluorescence microscopy with white laser technology [18, 19, 20], are expensive, complicated, need highly trained personal and not readily available especially in point of care places, for quick assessments or in remote areas.

In order to successfully guarantee appropriate safeguard and targeted treatment, as well as to prevent the needless administration of antibiotics, there is a need to develop rapid, accurate sensing techniques that are also cost‐effective and simple to use, in order to identify and detect pathogens prior to treatment. Depending on the envisioned application, the sensitivity must be controllable as reporting functionalities for integration into self‐reporting wound dressing should signal the onset of infection, i.e., the critical colonization threshold, rather than low bacteria counts, which can be cleared by the immune response. It is accepted that the critical colonization threshold in wounds refers to 10^6^ CFU/mL, above which infection is highly likely to occur [21].

Different detection methods that bypass the aforementioned drawbacks of conventional methods have been investigated. The use of nanomaterials [22] as sensing materials, such as nanocapsules [23, 24], nanoparticles [25, 26], and nanofibers [27, 28] have garnered attention and are being extensively researched. These methods have utilized, among others, toxins and enzymes produced or secreted by particular bacteria that are the analytes detected by reporter systems [29]. The sensing substrates can also be incorporated in biomedical devices such as coatings or wound dressings. This autonomous reporting capability enables prompt infection detection, hence making it appropriate for potential widespread future use [30].

This study expands on the previously reported use of colorimetric sensor substrates as reporter systems [30, 31, 32, 33, 34, 35]. This approach has not only proven to be rapid but also efficient in the detection of bacteria at relevant concentrations for infection detection [32]. Chromogenic or fluorogenic substrates are cleaved by characteristic enzymes and can be detected spectroscopically, by mobile phone cameras, or by bare eye detection [33, 35]. These sensoing approaches exploit color changes, which are caused by chemical reactions of the above mentioned types of sensing substrate and bacterial enzymes that are produced and secreted by those bacteria. β‐Gus, which is generated by 98% of all known *E. coli* strains, may serve as an exemplary target analyte.

The β‐Gus catalyzed reaction of 4‐methylumbeliferyl‐β‐D‐glucuronide hydrate produces a glycone unit and the fluorophore 4‐methylumbelliferone (4‐MU) [36]. The incorporation of the sensing dye into materials with increased surface‐to‐volume ratio, such as in chitosan/PEO‐based nanofibers, led to increased sensitivity. A study by Kaur et al. [28] on MUG fluorogenic functionalized nanofibers for the detection of *E. coli* demonstrated an enhanced sensitivity compared with neat chitosan hydrogels, with an up to 3.4‐fold increase in the initial enzymatic hydrolysis rate, hence resulting in a lower limit of detection. However, this increase was much smaller than anticipated based on the increased surface‐to‐volume ratio, indicating that hindered access of the enzyme to the reactive moieties may be compromised due to the fibers’ morphology and limited porosity.

This current study also utilized chitosan as a base material owing to the ease of covalently functionalizing the free amine units [37, 38]. Porous forms of chitosan are known and have received considerable attention, especially in biomedical applications, such as dermal drug delivery mechanisms [39], implants, and tissue scaffolds [40], due to their highly interconnected macroporous, high loading capacity, and elastic structures that facilitate the diffusion of molecules, which is key in drug delivery systems [41, 42].

Motivated by the previous research findings, we hypothesize that the surface‐to‐volume ratio of chitosan equipped with 4‐methylumbelliferyl‐β‐D‐glucuronide hydrate as a β‐Gus reporting functionality controls the sensitivity. In an attempt to further increase the sensitivity of such autonomously reporting sensing materials, porous forms of chitosan, namely hydrogel beads and freeze‐dried scaffolds, were fabricated, functionalized with a fluorogenic substrate MUG, and the sensitivity of the detection of β‐Gus and of β‐Gus produced by two strains of *E. coli* (MACH‐1) and NCTC 10418 was studied.

## 2 Material and Methods

### 2.1 Materials

The materials used in this study have been utilized in previously published work [28]: Chitosan (CS, medium molar mass, 190–310 kDa, 75%–85% deacetylated), *N*‐3(3‐dimethylaminopropyl)‐*N*‐ethylcarbodiimide hydrochloride (EDC.HCL, ≥99%), β‐glucuronidase from *E. coli* (β‐Gus, 694.3 units/mg, E.C. 3.2.1.31; type IX‐A, lyophilized powder, 25,000 units/g protein), phosphate saline buffer solution (PBS, pH 7.40), 4‐methyllumbelliferyl‐β‐D‐glucuronide (MUG), and *N*‐hydroxy succinimide (NHS) were purchased from Sigma‐Aldrich, Germany. Acetic acid (glacial, J. T. Baker, Germany), sodium hydroxide (NaOH, *>*98.8%, ChemSolute), disposable acrylic cuvettes (10 mm ×10 mm × 45 mm, Sarstedt, Germany), syringes (10 mL, Henke‐Ject, Germany), needles (21, Braun, Germany), Milli‐Q water (Millipore Advantage A10 system, Schwalbach, with Millimark Express 40 Filter, Merck, Germany), Lysogeny broth (LB) agar (yeast extract 5 g/L, tryptone 10 g/ L, NaCl 5 g/L, Luria/Miller, Carl Roth, Germany) and LB medium (Luria/ Miller, Carl Roth, Germany) were purchased as mentioned. LB agar and LB medium were sterilized at 121°C, 1.2 bar for 15 min in an autoclave (Systec VB‐150, Systec GmbH, Linden, Germany).

### 2.2 Fabrication of the Different Chitosan Morphologies

For film formation, CS solutions were prepared, as reported by Jia et al. [43] Briefly, 1.5% (w/v) chitosan solutions in 1% acetic acid were prepared by stirring overnight at 50°C. The solution was then suction filtered to remove impurities and particles (d ≥ 2.5 µm). For preparing films, 10 mL of the CS solution was deposited onto a glass petri dish (diameter 5.5 cm) and dried overnight in a flow hood. The film was peeled off and neutralized by immersing in NaOH solution (10 mL, 0.1 m) for 5 min, followed by washing thrice with copious amounts of Milli‐Q water and then drying in a stream of nitrogen.

The CS beads were fabricated as reported by Ren et al. [44] 20 mL of 1.5% (w/v) CS solution was delivered dropwise using a syringe pump (Aladdin syringe pump, 941‐371‐1003, World precision instruments, Florida, USA) with a blunt needle (0.80 mm × 22 mm) at a rate of 0.4 mL per minute to a beaker containing 100 mL of 1 m NaOH. This was done while stirring to prevent the clumping of the formed beads. The beads were then rinsed with Milli‐Q water until neutral. The collected beads were then transferred to a 50 mL round‐bottom flask and freeze‐dried (freezing using liquid nitrogen and drying using a vacuum freeze dryer).

The CS scaffolds were prepared as reported by Alizadeh et al. [45] 20 mL CS solution was added in a 50 mL round‐bottom flask and freeze‐dried (freezing using liquid nitrogen and drying using a vacuum freeze dryer). The scaffold was then neutralized using 0.1 m NaOH for 10 min, followed by rinsing 3 times with a copious amount of Milli‐Q water and dried overnight in a flow hood.

### 2.3 Characterization of CS Film, Beads and Scaffold

#### 2.3.1 Scanning Electron Microscopy (SEM)

The scanning electron microscope analysis was carried out in order to determine the surface morphology and topography of CS films, beads, and scaffolds [28]. The SEM images were acquired on gold sputtered samples using a CamScan 44 (Applied Beams, Beaverton) electron microscope with a working distance of 31 mm using an acceleration voltage of 25 kV and an imaging current of 2.33 A. The thickness of the CS film was also determined using SEM.

#### 2.3.2 Attenuated Total Internal Reflectance Fourier Transform Infrared (ATR‐FTIR) Spectroscopy

ATR‐FTIR spectra of the prepared CS film, beads, and scaffold were recorded on a Tensor 27 Fourier transform infrared spectrometer (Bruker Optik GmbH, Siegen, Germany) using a diamond ATR crystal [28]. The spectra were recorded in the 600–4000 cm^−1^ wavenumber range with a spectral resolution of 4 cm^−1^. Air was used to obtain a background spectrum.

#### 2.3.3 Fluorescence Spectroscopy

For the investigation of the apparent enzyme kinetics, a Varian Cary Eclipse spectrometer (Mulgrave, Victoria, Australia) was used [28]. The spectra were recorded using 1 cm path‐length acrylic cuvettes at 25°C at a scan rate of 120 nm/min and an excitation and emission resolution of 2.5 nm. For in vitro bacteria experiments, the fluorescence intensity was measured using a micro plate reader (Tecan Infinite M200, Tecan Austria GmbH, Grödig/ Salzburg, AUT). The measurements were recorded with fluorescence top reading mode with *λ*_ex_ = 365 nm and *λ*_em_ = 450 nm.

### 2.4 Modification of the CS Films, Beads, and Scaffolds

EDC/NHS chemistry was used to covalently couple fluorogenic substrate MUG onto the CS film, beads, and scaffold as reported in the literature [28, 33]. Briefly, 1 mm MUG was dissolved in a buffered solution (PBS, pH 7.40) in ambient atmosphere, followed by the addition of EDC (30 mol/mol of ─COOH) and NHS (30 mol/mol of ─COOH) while stirring. The solution was stirred for 1 h. The CS film, beads, and scaffold were immersed in this modification solution for 12 h while shaking moderately. The modified substrates were then washed with buffered PBS (solution replacement interval: 30 min, for 2 h) until no absorbance of free MUG was observed in the washing solutions of all substrates (Figure S1). The samples were then dried overnight under a laminar flow hood.

### 2.5 Enzymatic Hydrolysis of MUG‐Modified CS Film, Beads, and Scaffold

To afford consistent benchmarking, the hydrolysis was carried out as reported previously [28]: The enzymatic hydrolysis of the MUG‐modified film, beads, and scaffold was adapted from literature [28, 46]. Briefly, 5.0 mg of dried samples was added to an acrylic cuvette, followed by an addition of 3.0 mL of enzyme solutions prepared in PBS (pH 7.40) with varied concentrations. The fluorescence scan was recorded with a Varian Cary Eclipse spectrometer at *λ*_ex_ = 365 nm with 5 min intervals for 3 h after mixing the solution in a cuvette within each measurement scan using a magnetic stirrer. For measurement, 1.50 mL of solution was taken in another cuvette, the fluorescence was recorded, and the solution was transferred back to the original cuvette containing the sample. The kinetics data were obtained for 3 different enzyme concentrations (50, 100, and 200 nm) to observe the effect of enzyme concentration on the initial apparent reaction rates. As blank, the modified samples were measured in PBS (pH 7.40).

### 2.6 Determination of Limit of Detection (LOD) and Limit of Quantification (LOQ)

A 4‐MU standard curve in PBS (pH 7.40) was prepared to calculate the LOD and LOQ of 4‐MU. A Varian Cary Eclipse Fluorescence spectrometer was used to detect the fluorescence emission intensities of various 4‐MU concentrations in PBS at λ_ex_ = 365 nm. The values of the LOD and LOQ were calculated as reported previously [28].

### 2.7 Bacteria Experiments for β‐Gus Detection, In Vitro

Non‐pathogenic *E. coli* MACH1‐T1 (Invitrogen, California, USA), here noted as MACH‐1, and pathogenic *E. coli* NCTC 10418, here noted as NCTC, were used in this study, aligned with the previously published protocol at a final concentration of 10^8^ CFU/mL [28]. 1.00 mg of dried MUG‐modified CS films, beads, and scaffolds were analyzed in the plate reader (*λ*_ex_ = 365 nm, *λ*_em_ = 450 nm) with a measurement repeat of 10 min. MUG‐modified samples and the neat bacteria strains in LB medium served as controls. All data were recorded in triplicate.

## 3 Results and Discussion

Chitosan films were prepared using drop casting, while the beads and scaffold were prepared via freeze‐drying. The morphology and topography of each substrate were characterized by SEM. The fluorogenic substrate MUG was covalently grafted onto chitosan for each morphology using EDC/NHS chemistry to obtain β‐Gus sensitive CS substrates. The functionalized CS substrates were then characterized using ATR‐FTIR spectroscopy. The hydrolysis of the modified films, beads, and scaffolds was investigated in solution containing β‐Gus or in bacterial suspensions. The impact of increased surface area through freeze drying on the sensitivity was investigated by comparing the performance of the three different CS morphologies.

### 3.1 Characterization of CS Film, Beads, and Scaffold

**FIGURE 1:**
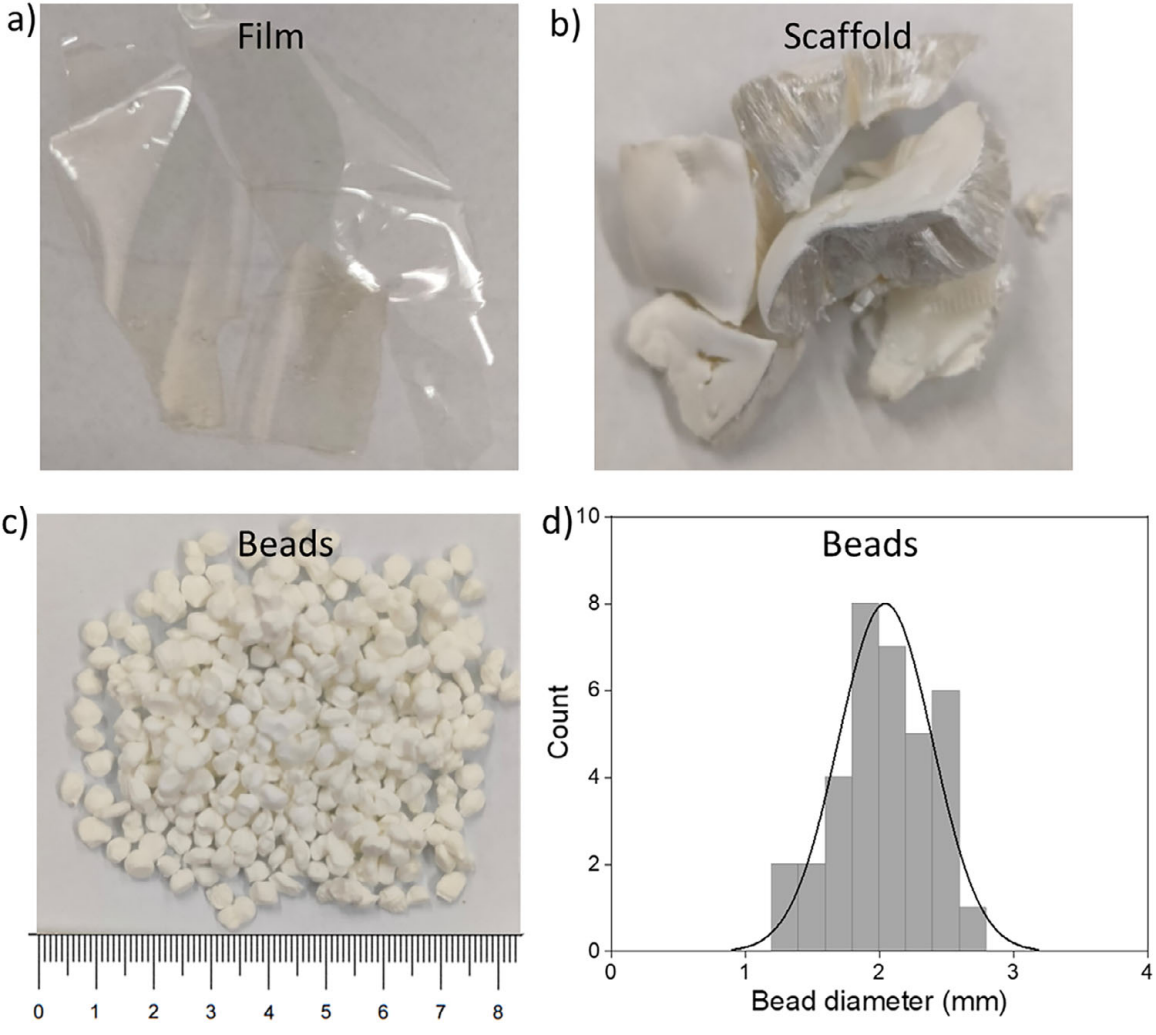
Photographs of the CS (a) film, (b) scaffold, (c) beads (scale in cm), and (d) histogram of the bead diameter.

Figure 1 shows the photographs of the CS film, scaffold, and beads. The surface areas of the CS film and beads were estimated using their geometric measurements. CS film thickness, as determined by SEM, was 24.9 ± 8.4 µm. The CS bead size was determined using calipers, with measurements obtained from 35 individual beads. The mean bead diameter was calculated to be 2.0 ± 0.4 mm, and the corresponding CS bead diameter distribution histogram is shown in Figure 1d. The surface area of a single bead was calculated to be 13.1 mm^2^. For 5.00 mg sample comprising 14 beads, the total surface area of the beads was 184 mm^2^. In comparison, for CS film of the same mass, a surface area of 7.0 mm^2^ was determined by measuring the dimensions of the sample.

**FIGURE 2:**
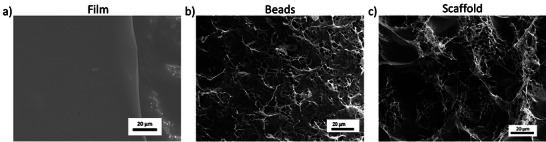
SEM micrographs of the surfaces of (a) films, (b) beads, and (c) scaffolds.

The data of morphology analyses via SEM of the three CS substrates are shown in Figure 2. It can be observed that the CS beads and scaffold exhibit pores as compared to the film under similar magnifications.

It is well established that during freezing, which takes place under the crystallization point of water, ice crystals are formed, which play a vital role in pore formation during drying. The ice crystals undergo a sublimation process in a vacuum, creating voids within the structure [41, 44, 47]. In this study, the samples were freeze‐dried under vacuum for 24 h. It was noticed that the CS beads had a compact, small pore structure, while the scaffold had large pores with more interconnectivity. The CS beads were formed through acid‐base neutralization as the CS solution was delivered as droplets using a syringe in 1 m NaOH. Without dissolution, the beads hardened, remaining in a spherical shape. It is presumed that this shape limited the size of the ice crystals that were formed within the bead matrix. This resulted in smaller‐sized discrete pores that restricted effective diffusion of reactants into the bead's interior. In contrast, the scaffold was frozen in liquid form in a round‐bottomed flask, and the ice crystals had more space for extensive ice nuclei growth, resulting in sponge like material with larger heterogeneous interconnected pores that enhanced the diffusion of reactants.

**FIGURE 3:**
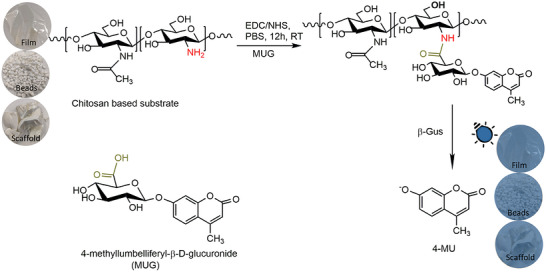
Schematic of the wet chemical functionalization of chitosan film, beads, and scaffold substrates with the fluorogenic substrate MUG using standard EDC/NHS coupling. Upon enzymatic cleavage of the enzyme‐labile bond, the blue‐colored dye 4‐methyllumbelliferone (4‐MU) is liberated.

β‐Gus sensing CS film, beads, and scaffold were obtained by grafting the fluorogenic enzyme sensing substrate through the formation of an amide bond between the primary amine group of the glucosamine unit of chitosan and a carboxyl group of the MUG via EDC/NHS chemistry (Figure 3).

**FIGURE 4:**
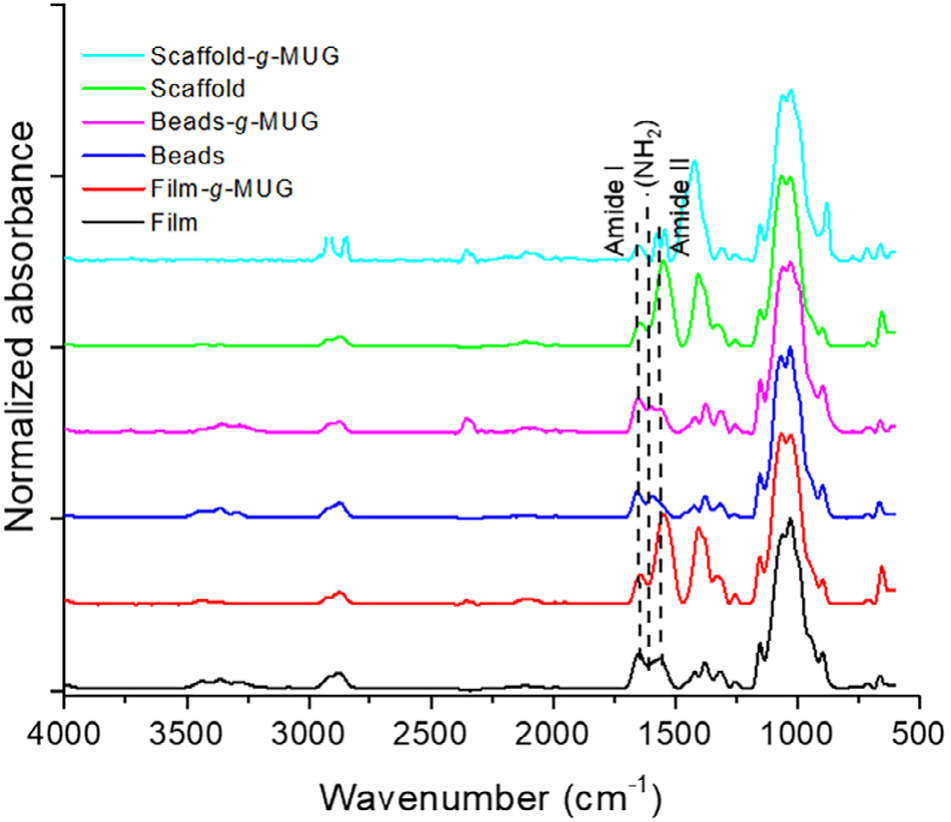
ATR‐FTIR spectra of non‐modified and MUG‐modified CS films, beads, and scaffolds.

The MUG‐functionalized CS substrates were then characterized using ATR‐FTIR spectroscopy. Figure 4 shows the normalized absorbance spectra. This rescaled the intensity values relative to the maximum signal, which was at 1031cm^−1^ to allow direct comparison among relative band intensities of non‐modified CS film, beads, and scaffold, and the MUG modified films, beads, and scaffolds. The main vibration bands of focus were the amide II (N─H), primary amine (NH_2_), and amide I(C═O), which were observed at around 1550, 1599, and 1650 cm^−1^, respectively, for the unmodified film, and 1555, 1605, 1658 cm^−1^ for the unmodified beads, and 1550, 1600, 1648 cm^−1^ for the unmodified scaffolds. The assignment of the important vibration bands of each of the modified substrates is summarized in Table S1. It was observed that the band intensity of the ‐NH_2_ band in the modified substrates disappeared, indicating free amine groups participated in the coupling with the carboxyl group of MUG. The coinciding appearance of amide I and amide II bands further indicates the amide bond formation.

### 3.2 Enzymatic Hydrolysis of MUG‐Grafted CS Film, Beads, and Scaffold

**FIGURE 5:**
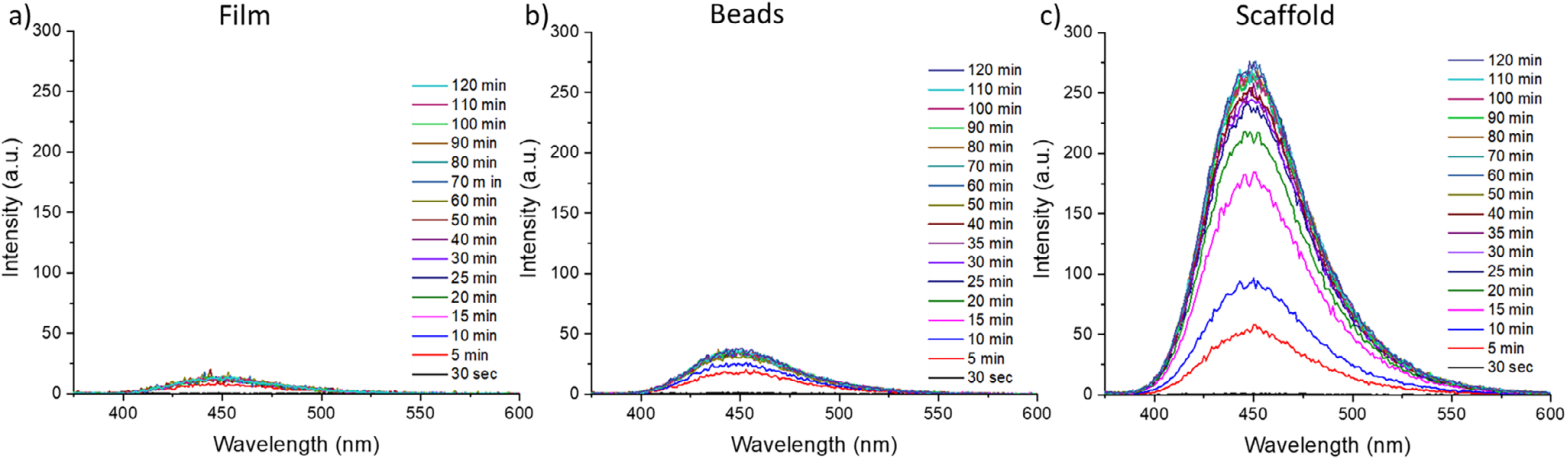
Fluorescence spectra acquired during the enzymatic reaction of 5 mg of MUG‐modified CS (a) film, (b) beads, and (c) scaffold in 200 nm β‐Gus at λ_ex_ = 365 nm.

The enzymatic hydrolysis of the MUG‐modified films, beads, and scaffold was carried out in buffered β‐Gus enzyme solutions in phosphate buffer solution (pH 7.40) under ambient conditions. These reactions result in the release of the coumarin derivative 4‐MU. The reaction was evaluated through sequential measurements using fluorescence spectroscopy. The presence of free deprotonated 4‐MU was observed at an emission wavelength of 450 nm, which continually increased with time. A typical kinetic measurement of the films, beads, and scaffolds in solutions of 200 nm β‐Gus recorded over time is shown in Figure 5. To confirm the signal is generated solely due to the enzymatic cleavage of covalently bound MUG, the fluorescence spectrum of the non‐modified sample in the presence of the enzyme, and the modified sample in the presence of PBS was also recorded as controls (Figure S2). No fluorescence signal of released 4‐MU was observed in the control experiment.

Under similar conditions of reaction, the modified film, beads, and scaffold exhibited different signal intensities. The scaffold demonstrated the highest intensity, followed by the beads and finally the film. The reaction initially occurs at the surface of the substrates and then proceeds to take place inside the pore network of the substrates. The beads and scaffold, which are porous, facilitate the diffusion of the enzyme into the high surface area porous system, hence resulting to higher intensities as compared to the film.

**FIGURE 6:**
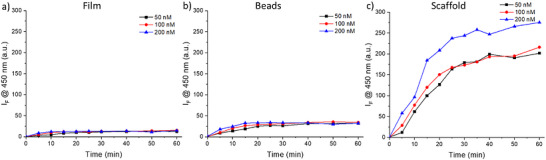
Fluorescence kinetics plots of the MUG‐modified (a) film, (b) beads, and (c) scaffold in different concentrations of β‐Gus at λ_ex_ = 365 nm.

Figure 6 shows the kinetic plot of the increasing fluorescence emission of 4‐MU for the first 60 min of the reaction of modified films, beads, and scaffolds in experiments with an enzyme concentration of 50, 100, and 200 nm, respectively.

An increase in β‐Gus concentration resulted in enhanced release of 4‐MU. The plots are characterized by a plateau that was reached within < 60 min. The enzymatic reaction was carried out for 120 min with measurements taken every 10 min, until no further increase in intensity was observed (Figure S3). The higher enzyme concentration facilitated a faster cleavage of MUG to 4‐MU compared to lower enzyme concentrations in the same timeframe. This implies that, at higher enzyme concentrations, there are more available enzyme sites, hence adequate contact with the substrate compared to lower enzyme concentrations. As long as the enzyme concentration remains a limiting factor of the reaction, this remains applicable. Initially, sufficient substrate is available to bind with the enzyme's active sites, however, as the reaction progresses and the substrate is used up, a further increase in enzyme concentration does not enhance 4‐MU production. The amount of released 4‐MU after enzymatic reaction in the presence of different enzyme concentrations can be determined by using a standard curve of 4‐MU in PBS (pH 7.40) (Figure S4). The corresponding 4‐MU concentrations after 120 min reaction are summarized in Table S2. It was noted that in the presence of 200 nm β‐Gus, the film, beads, and scaffold released 4‐MU to result in concentrations of 125.2, 453.2, and 3388 nm of 4‐MU, respectively. This significant difference in the released 4‐MU under identical reaction conditions implies that the scaffold offers a larger surface area, thereby supporting a faster enzymatic reaction rate. Another important factor to consider for enzymatic response is the degree of functionalization of MUG to different chitosan morphologies, as the larger surface area in the scaffold could offer higher MUG loading. Further work on the quantification of MUG could decouple the effects of substrate loading on enzymatic response.

**FIGURE 7:**
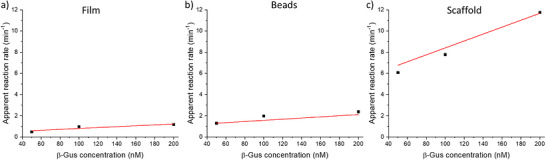
Plots of the initial apparent reaction rate for different concentrations of (a) films, (b) beads, and (c) scaffolds. Linear least square fits of the data afforded intercept values of 0.375 ± 0.246, 1.09 ± 0.322, and 4.08 ± 0.195 min^−1^ for the film, beads, and scaffold, respectively.

Figure 7 shows how a variation of the enzyme concentration alters the initial apparent rate of all types of samples. The initial apparent reaction rates were determined by applying a linear least‐squares fit to the kinetics data obtained during the first 10 min of the enzymatic reaction.

The initial apparent reaction rate was observed to increase with the enzyme concentration. This trend is consistent with Michaelis‐Menten kinetics [48, 49]. The resulting initial apparent reaction rates for the modified CS film, beads, and scaffold are summarized in Table S3. For 200 nm β‐Gus, the initial apparent rates were found to be 1.17 ± 0.28 min^−1^ for the film, 2.37 ± 0.62 min^−1^ for the beads, and 9.54 ± 1.08 min^−1^ for the scaffold substrate. Thus, the MUG‐modified scaffold exhibited an enzyme hydrolysis rate up to 8 times higher than that of the film and 4 times higher than that of the beads. Similarly, the MUG‐modified beads showed a 2 times faster hydrolysis rate than the modified film. The substantially higher rate observed for the scaffold compared to films and beads can be attributed to its higher surface‐to‐volume ratio and three‐dimensional porous architecture, which ensures maximum exposure of reactive sites. In contrast, the films provide only a planar surface, while diffusion limitations within the beads might restrict enzyme accessibility to internal reactive sites. The interconnected, open network of the scaffold minimizes such diffusion barriers, resulting in the improvement of the catalytic efficiency by 8‐fold compared to films and 4‐fold compared to beads.

Comparing surface area and reaction rates between beads and film, the beads exhibited a 26‐fold larger surface area; however, the corresponding hydrolysis rate increased by only twofold. This modest enhancement indicates that a considerable portion of the bead surface was kinetically inaccessible, likely due to diffusion constraints or the steric inaccessibility of pores. As a result, only a fraction of the available surface contributed effectively to catalysis, indicating partial utilization of the total surface area.

The enhanced sensitivity factor may also be contributed by the swelling of chitosan that has been established to have a ratio of about 6 to 7 [50, 51]. This swelling, combined with porous morphology, facilitates improved enzyme accessibility to the MUG‐conjugated chitosan matrix. Overall, modifying the substrate morphology has an impact on the surface area to volume ratio [45], providing a strategic approach to enhance sensor response.

Moreover, the enzymatic hydrolysis behavior observed in this study can be compared with previously reported chitosan‐based hydrogel systems. For instance, Jia et al. developed β‐Gus enzyme‐responsive chitosan hydrogels and reported enzymatic reaction rates of 2.5 × 10^−3^ min^−1^ (within the first 30 min) and 1.1 × 10^−4^ min^−1^ (within the first 40 min) for chitosan conjugated with 5‐bromo‐4‐chloro‐3‐indolyl‐b‐D‐glucuronide (X‐Gluc) and 4‐nitrophenyl‐b‐D‐glucuronide (PNPG), respectively [33], in the presence of 1 mm β‐Gus. In a subsequent study, these authors reported increased initial apparent rates of 3.29 × 10^−3^ and 2.50 × 10^−3^ min^−1^ (t = 30 min) using 2 mm β‐Gus for chitosan hydrogel samples of various shapes that had been functionalized with different degrees of substitution of chromogenic substrate X‐Gluc [52]. In this and a related study [43], the enzymatic hydrolyses proceeded at relatively slower rates, despite the use of significantly higher enzyme concentrations to accelerate the hydrolysis process. Furthermore, Kaur et al. reported the development of chitosan‐based nanofibers for enhanced β‐Gus detection, demonstrating an enzymatic reaction rate of 3.03 ± 0.38 min^−1^ (t = 15 min) with 200 nm β‐Gus [28]. This rate was higher than that observed for the CS beads in the present study but lower than that achieved with the scaffold substrates.

### 3.3 LOD of Enzyme β‐Gus

**FIGURE 8:**
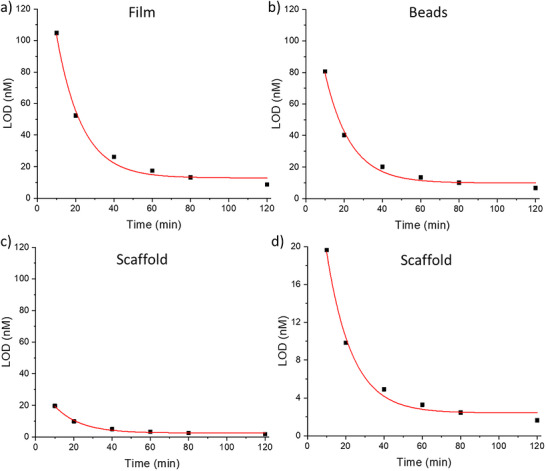
The limit of detection vs. hydrolysis time for CS (a) films, (b) beads, and (c) scaffolds. Plot (d) shows a zoomed image of plot (c) to show the significant difference in LOD value. The data was fitted with an exponential fit.

The LOD for the detection of ß‐GUS was analyzed as reported previously, see Figure 8 [28].

The LOD of β‐Gus was observed, as expected, to decrease with reaction time. After 60 min, the LOD were observed to be 17.5 nm for the MUG‐modified film, 13.4 nm for the beads, and 3.3 nm for the scaffolds. These results validate that the scaffold and bead morphologies report the presence of β‐Gus more sensitively than the films. Kaur et al. [28] reported for the same observation time an LOD for β‐Gus of 16 nm for CS‐PEO hydrogel films and 4.7 nm for hybrid CS‐PEO nanofibers. This trend supports the conclusion that LOD improves with increasing surface area or by altering the morphology of the chitosan matrix. Similar findings were reported by Kinyua et al. [50], who investigated the influence of surface area using chromogenic substrates. For an observation time of 60 min, they observed β‐GUS LODs of 25 and 13 nm for CS‐PEO hydrogel and CS‐PEO nanofiber, respectively, further corroborating the morphology‐sensitivity relationship.

Jia et al. also reported LODs for β‐Gus detection in a study of chitosan hydrogels functionalized with chromogenic substrates. For an observation time of 60 min, LODs of ≤ 5 nm [52] and a LOD of 4.5 nm [43] were observed. In these studies, thin hydrogel films on solid substrates were studied that were functionalized after film formation with a chromogenic dye. Notably, the enzyme concentrations in those experiments were higher than those in the current study. Therefore, the current findings demonstrate that lower LODs area attainable at lower enzyme concentrations, highlighting the advantage of morphology‐driven sensitivity enhancement.

### 3.4 In Vitro Detection of β‐Gus

**FIGURE 9:**
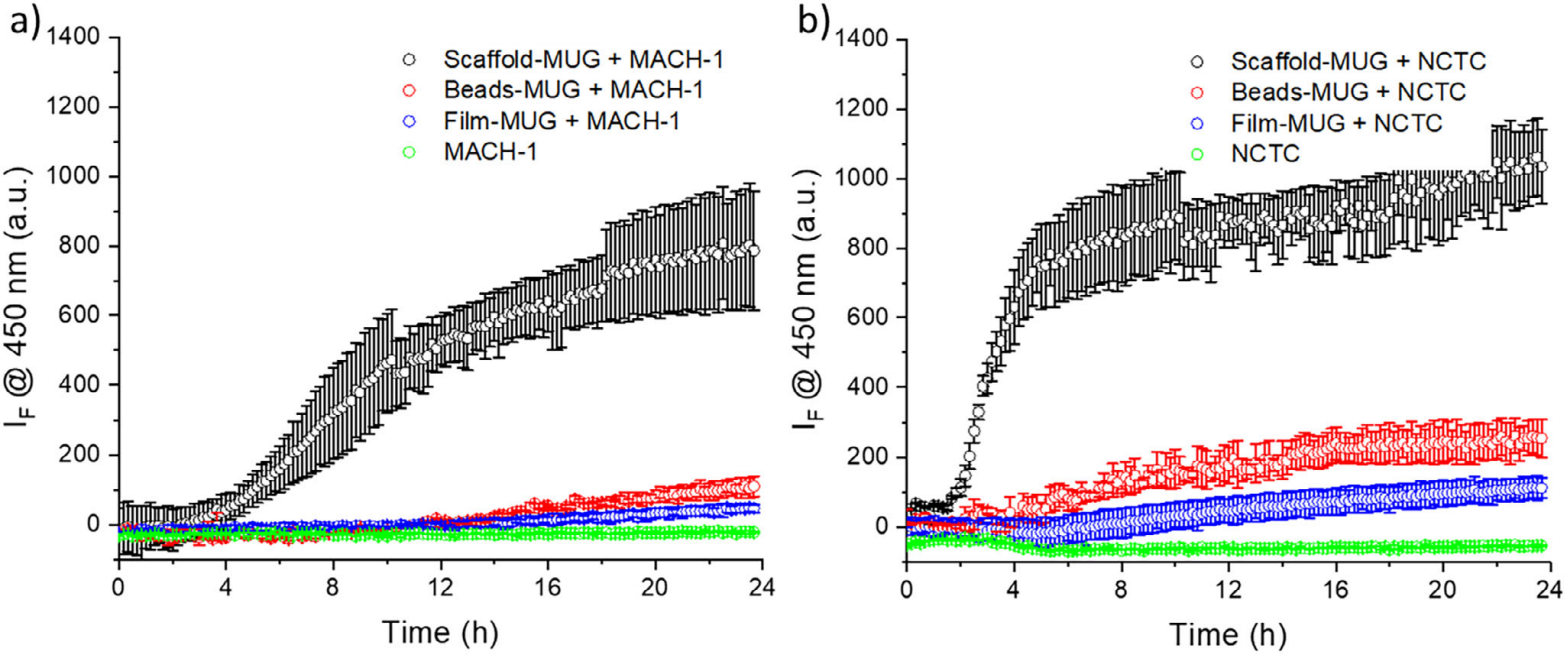
Fluorescence kinetic plots of: (a) MUG‐modified film, beads, and scaffold in the presence of *E. coli* MACH‐1 and (b) in the presence of *E. coli* NCTC strains studied for 24 h at λ_ex_ = 365 nm with measurements every 10 min.

Finally, the sensing CS films, beads, and scaffolds were tested in vitro in the presence of *E. coli* bacteria suspensions. More than 98% of known *E. coli* are known to secrete the β‐Gus enzyme, which plays a huge role in bacterial metabolic activities [53]. During this study, both pathogenic *E. coli* (NCTC 10418) and non‐pathogenic *E. coli* (MACH‐1) strains were investigated. Figure 9 shows the results for the three types of morphologies are shown, both for MACH‐1 and NCTC bacteria.

The fluorescence intensity recorded at 450 nm remains relatively constant, independent of time for both bacterial strains in LB medium. By contrast, a steady increase in fluorescence intensity is observed for both bacterial strains that contain MUG modified films, beads, and scaffolds. This observation demonstrates that the amount of released 4‐MU in the medium increases as the β‐Gus enzyme produced by the bacteria strains cleaves the β‐glycosidic linkage on the MUG coupled films, beads, and scaffolds. So the β‐Gus detection signal depends on bacterial density and enzyme release. As previously observed in the enzymatic reactions, the fluorescence intensity of the scaffold is higher, followed by that of the beads, and finally the film, verifying that both the scaffold and beads are more sensitive than the film morphology. Among both bacterial strains, higher fluorescence signal intensities were observed for the NCTC as compared to the MACH‐1 strain, while no changes in emission were observed for the controls (Figure S5). The bacterial growth in the presence of modified samples was assessed by measuring OD at 600 nm. The growth kinetics for the different modified samples, corrected for the absorbance for modified samples containing bacterial strains, is shown in Figure S6. The modified scaffold shows higher absorbance in LB as compared to modified beads and film (Figure S7), due to its larger surface area, which enhances the light scattering.

β‐Gus from *E. coli* is predominantly an intracellular enzyme. Hence, after cell lysis or for high bacterial densities, an enhanced sensor response is expected [54]. Furthermore, β‐Gus expression is known to peak during the stationary phase of bacterial growth [54], necessitating these conditions for effective sensor performance. This accounts for the initial lag phase observed in the detection signal (Figure 9), which was approximately 4 h for MACH‐1 and 2 h for NCTC strains. The modified scaffold showed significantly enhanced sensitivity‐ ∼7 times higher for MACH‐1 and 10 times higher for NCTC compared to modified films. These results demonstrate the potential applicability of the scaffold‐based system for the detection of *E. coli* producing β‐Gus. The current system does not differentiate between pathogenic and non‐pathogenic strains, but it provides a rapid indication of bacterial presence. Although β‐Gus is not exclusively specific to *E. coli*, its elevated activity under infection conditions makes it a useful biomarker for early‐stage detection. Future developments will focus on improving specificity by integrating additional and more selective biomarkers. Furthermore, to enhance biological relevance, subsequent studies will aim to establish correlations between enzyme activity and bacterial load, enabling sensitivity to be expressed directly in CFU/mL.

The CS scaffold‐based enzyme sensing substrate developed in this study exhibited a significantly lower LOD compared to both the beads and film morphologies, and also surpassed the performance of previously reported chitosan‐based β‐Gus sensing systems. Further optimization of parameters, such as freezing temperature and chitosan concentration, could enhance scaffold characteristics such as mechanical properties, surface area, and porosity, thereby improving sensitivity even further [55, 56]. Moreover, chitosan‐based scaffolds can also be easily fabricated on a large scale and easily incorporated into biomedical devices, making them promising candidates for practical biosensing applications [57, 58].

## 4 Conclusion

Chitosan‐based films, beads, and scaffold were successfully functionalized with the fluorogenic substrate MUG and evaluated for their performance in enzymatic β‐Gus reactions and in vitro bacterial detection. The scaffold showed enhanced sensitivity compared to the beads and film. Among the investigated morphologies, the freeze‐dried chitosan scaffold exhibited the highest sensitivity toward β‐Gus, outperforming both the film and beads. At 200 nm enzyme concentration, the scaffold showed 8‐times higher initial apparent reaction rate than the film and a 4‐times increase relative to the beads. After 60 min, the LOD were 3.3 nm for the scaffold, 13.4 nm for the beads, and 17.5 nm for the film, underscoring the relationship between surface‐to‐volume ratio, porosity, and sensing performance. Consistent in vitro results with both pathogenic and nonpathogenic *E. coli* strains further validated the superior sensitivity and responsiveness of the scaffold‐based system. Owing to their biocompatibility, scalability, and tunable porosity, chitosan scaffolds hold strong potential for integration into autonomous biosensing platforms and biomedical diagnostic devices for early and selective bacterial infection detection.

## Funding

Financial support by Key Action 1 (KA1) of the Erasmus+ KA 171 project with Kenya, as well as Dedan Kimathi University of Technology, Kenya, and the University of Siegen is gratefully acknowledged.

## Conflicts of Interest

The authors declare no conflicts of interest.

## Supporting information




**Supporting File**: mabi70212‐sup‐0001‐SuppMat.pdf.

## Data Availability

The data that support the findings of this study are available from the corresponding author upon reasonable request.
