# Supplementary material for: Morphology‐Dependent Sensitivity of Chitosan‐Based Sensing Materials for the Colorimetric Detection of β‐Glucuronidase From Pathogenic E. coli
Source: Macromol Biosci. 2026 Jul 8;26(7):e70212. doi: 10.1002/mabi.70212 (PMC13346512; doi:10.1002/mabi.70212)
Supplement: Supplementary file 1 — Supporting File: mabi70212‐sup‐0001‐SuppMat.pdf. [file MABI-26-e70212-s001.pdf]

Supporting Information

Morphology-Dependent Sensitivity of Chitosan-Based Sensing Materials for the Colorimetric Detection of β-Glucuronidase from Pathogenic *E. coli*

Kawaljit Kaur,[#,1] Kinyanjui Stephanie,[#,1,2] Nthiga Esther,[2] Douglas Onyancha,[2] Holger Schönherr[1]*

1. Physical Chemistry I, Research Center of Micro- and Nanochemistry and (Bio) Technology, Department of Chemistry and Biology, School of Science and Technology, University of Siegen, 57076 Siegen, Germany
2. Department of Chemistry, Dedan Kimathi University of Technology. Nyeri-Mweiga Road Nyeri 10143, Kenya

- Correspondence: schoenherr@chemie.uni-siegen.de

[#]: These authors contributed equally to the study.

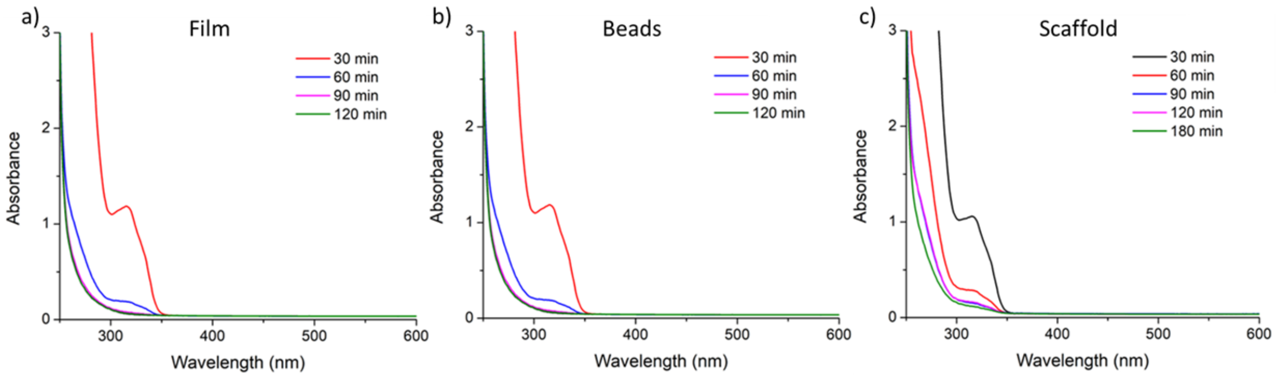

Figure S1: UV-Vis spectra of the MUG-modified CS a) film, b) beads, and c) scaffold after washing with PBS at pH 7.4 at interval times of 30 min. 1.5 mL of the washing solution was placed in an acrylic cuvette and the absorbance was measured using a Varian Cary 50 UV-Vis spectrophotometer.

Table S1: Band assignment of important functional groups observed using ATR-FTIR spectroscopy of modified and non-modified CS films, beads and scaffolds.

Assignment	Band position (cm^{-1}) Reference	Band position (cm^{-1}) Non-modified			Band position (cm^{-1}) Modified		
	Chitosan[1]	Film	Beads	Scaffold	Film	Beads	Scaffold
Amide II (N-H)	1550 – 1565	1550	1555	1550	1543	1555	1542
NH_2	1590 – 1610	1599	1605	1600	-	-	-
Amide I (C=O)	1620 – 1655	1650	1658	1648	1647	1644	-

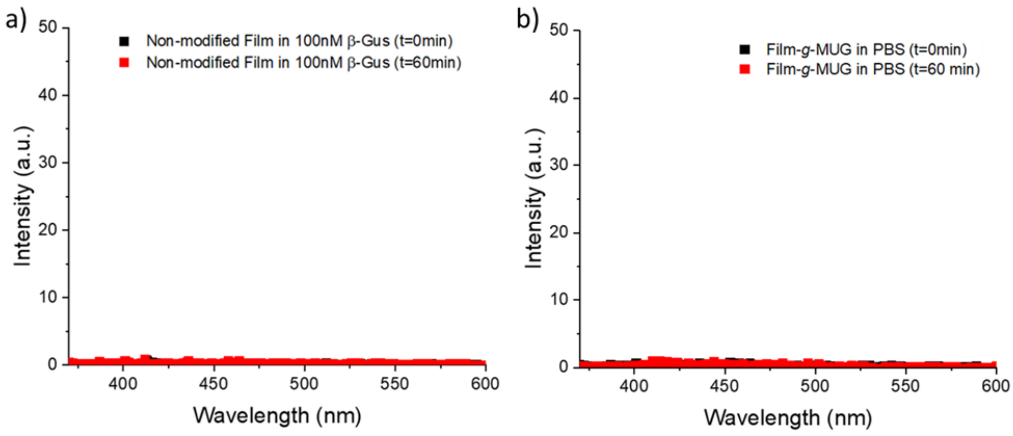

Figure S2: Fluorescence spectra of the enzymatic reaction of 5 mg of a) non-modified CS film in the presence of 100 mM β-Gus, and MUG-modified CS film in PBS recorded at an λ_{ex} = 365nm and with reaction time 0 min and after 60 min.

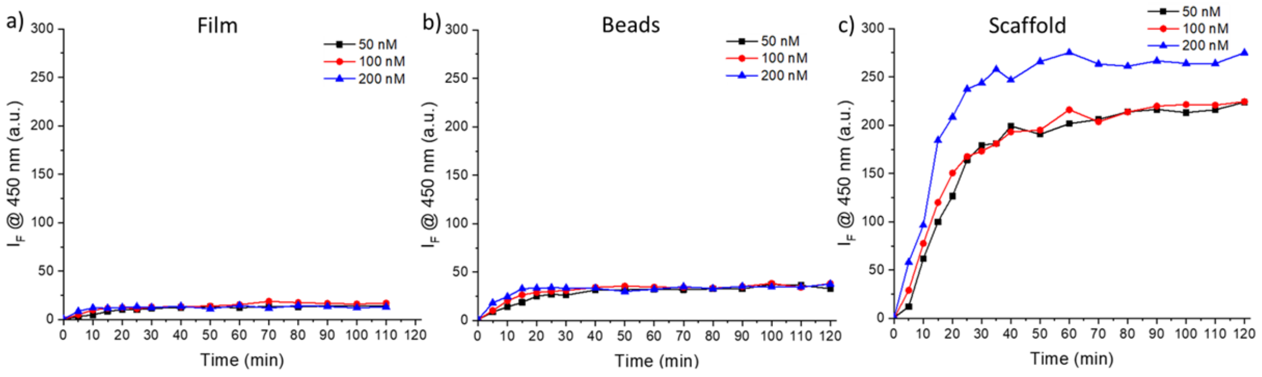

Figure S3: Fluorescence kinetics plots for MUG-modified CS a) film, b) beads and c) scaffold in the presence of β-Gus at different concentrations in buffer solution (pH: 7.4) studied for 120 min at λ_{ex} = 365 nm.

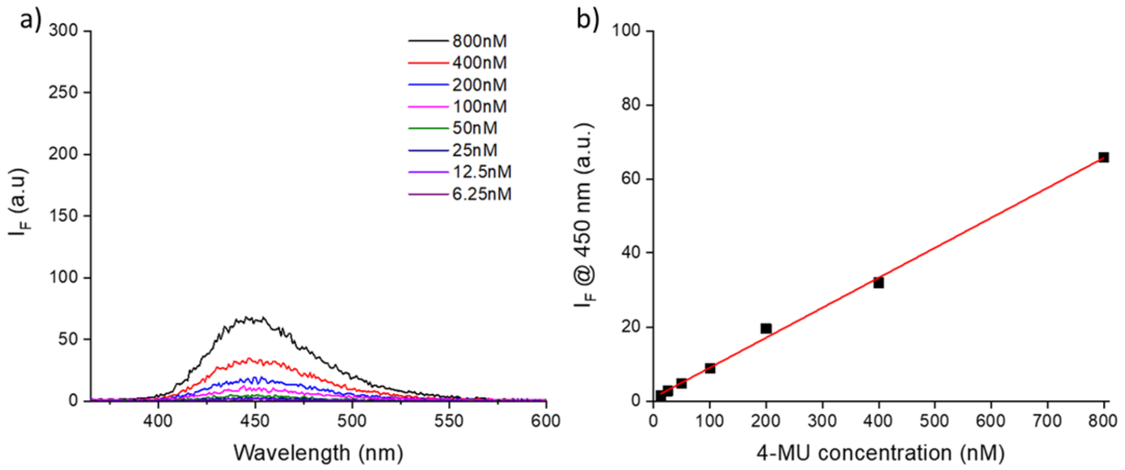

Figure S4: a) Fluorescence spectra of 4-MU measured at λ_{ex} = 365 nm for different concentrations in PBS (pH 7.40) b) calibration curve of 4-MU in PBS (pH 7.40). The data was fitted with a linear least square fit.

Table S2: The concentration of 4-MU released after 120 min of enzymatic hydrolysis of modified CS film, beads and scaffold studied with different enzyme concentrations.

β-Gus concentration (nM)	Released 4-MU (nM)		
	Modified film	Modified beads	Modified scaffold
50	157.8	394.1	2755
100	191.2	459.5	2763
200	125.2	453.2	3388

Table S3: The initial apparent reaction rates observed at different concentration of β-Gus after the enzymatic hydrolysis of the modified CS film, beads and scaffold.

β-Gus concentration (nM)	Initial apparent reaction rate (min^{-1})		
	Modified film	Modified beads	Modified scaffold
50	0.48±0.068	1.3±0.15	6.1±2.2
100	0.96±0.0076	2.0±0.040	7.7±1.1
200	1.2±0.28	2.4±0.62	9.5±1.1

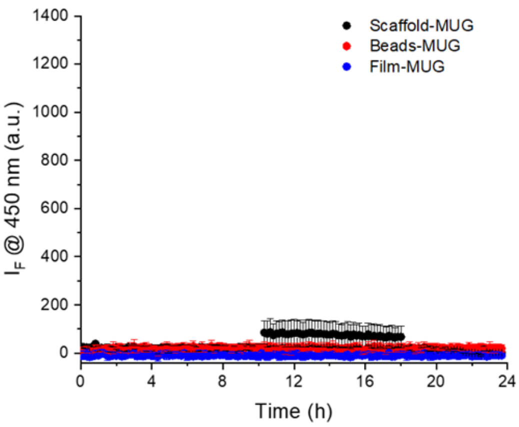

Figure S5: Fluorescence kinetic plots of modified CS a) scaffold, beads and film in LB medium measured at λ_{ex} = 365 nm with repeated measurements after 10 min. Fluctuations in the intensity was observed for the scaffold between 10-18 h resulting in slightly higher intensity values in that range.

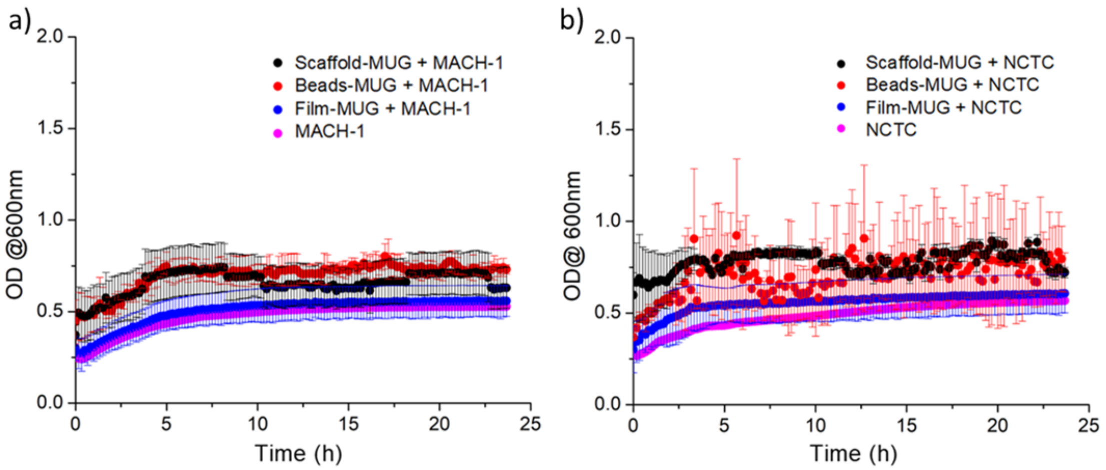

Figure S6: Growth kinetics plot of a) E. coli MACH-1 and b) E. coli NCTC in the presence of MUG-modified CS film, beads and scaffold studied for 24 h at an OD of 600 nm with measurement repeat 10 min.

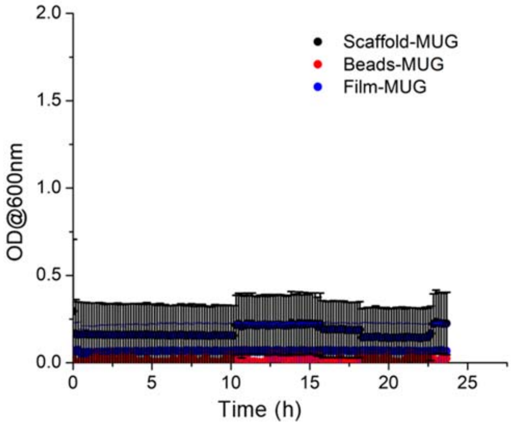

Figure S7: Absorbance plot of MUG modified CS film, beads and scaffold in the presence of LB medium studied for 24 h at an OD of 600 nm with measurement repeat 10 min.

Reference

[1] M.M.S. Ebrahimi, H. Schönherr, Enzyme-sensing chitosan hydrogels, Langmuir 30 (2014) 7842. https://doi.org/10.1021/la501482u.
